# CRNet: a multimodal deep convolutional neural network for customer revisit prediction

**DOI:** 10.1186/s40537-022-00674-4

**Published:** 2023-01-03

**Authors:** Eunil Park

**Affiliations:** 1 Department of Interaction Science, Sungkyunkwan University, Seoul, Republic of Korea; 2 Department of Applied Artificial Intelligence, Sungkyunkwan University, Seoul, Republic of Korea; 3 Department of Human-Artificial Intelligence Interaction, Sungkyunkwan University, Seoul, Republic of Korea

**Keywords:** Customer revisit, CRNet, MFDRD, Customer repurchase

## Abstract

Since mobile food delivery services have become one of the essential issues for the restaurant industry, predicting customer revisits is highlighted as one of the significant academic and research topics. Considering that the use of multimodal datasets has gained notable attention from several scholars to address multiple industrial issues in our society, we introduce CRNet, a multimodal deep convolutional neural network for predicting customer revisits. We evaluated our approach using two datasets [a customer repurchase dataset (CRD) and mobile food delivery revisit dataset (MFDRD)] and two state-of-the-art multimodal deep learning models. The results showed that CRNet obtained accuracies and Fi-Scores of 0.9575 (CRD) and 0.9436 (MFDRD) and 0.9730 (CRD) and 0.9509 (MFDRD), respectively, thus achieving higher performance levels than current state-of-the-art multimodal frameworks (accuracy: 0.7417–0.9012; F1-Score: 0.7461–0.9378). Future research should aim to address other resources that can enhance the proposed framework (e.g., metadata information).

**Supplementary Information:**

The online version contains supplementary material available at 10.1186/s40537-022-00674-4.

## Introduction

With rapidly advanced information and communication technologies, mobile applications are being used with ease. Mobile applications provide easy access to information and utilitarian values of online services for customers [1]. Moreover, recent developments and the distribution of mobile devices and technologies have improved industries. For instance, several restaurants provide services to customers inside the restaurant and via door-to-door delivery. In addition, owing to the COVID-19 pandemic, food delivery services have rapidly improved and diffused in several nations [2].

To minimize the potential contagion of the SARS-CoV-2 virus [3], most of the nations imposed stay-at-home policies and firmly recommended the use of mobile food delivery services (MFD services). This has also been applied in South Korea—a nation with advanced delivery services [4].

This also relates to restaurants’ survival, who need to respond to the huge demands of MFD services. Considering the findings of and definition provided by prior research on delivery services [5], MFD services are defined as “*food ordering/selecting/delivering systems, which connect specific restaurants, customers, and payment services through mobile applications*” [5, 6]. During this pandemic, the global market for MFD services has increased significantly. For instance, the global online food delivery markets in 2021 are estimated at approximately 127 billion USD, with 10% annual compound growth rate of approximately 115 billion USD.^1^

In line with this, a number of scholars have investigated customer behavior in terms of both the motivations for and hindrances of employing online food delivery services. Yeo et al. [7] considered 224 customers to explore their motivations for using online food delivery services by considering hedonic factors. Furthermore, Suhartanto et al. [8] investigated customer loyalty to online food delivery service providers by considering the perceived quality of the services. However, they were limited to addressing customers’ real service repurchases with several limitations–including the difficulties of data securement.

To efficiently address this issue, several scholars employed data mining or machine learning approaches for customer revisit behavior [9]. As one of the representative examples, Nilashi et al. [10] employed text mining, clustering and predictive regression techniques for customer decision making procedures via 4461 online review comments of 35 vegetarian friendly restaurants. However, the majority of prior examples employed single modality in addressing customer revisits (e.g. text [11]). Recent approaches indicated that employing multimodalities can contribute to the better understanding of customer-related tasks in several domains (e.g. healthcare [12], social media [13]).

To address this, we propose CRNet, a multimodal deep convolutional neural network for predicting customer revisits in MFD services. “Related work” section presents several cornerstones of data-driven approaches for customer revisits. “Our approach” section discusses the experiment conducted using the collected dataset. Both the results and implications are presented in “Experimental setup” and “Experimental results” sections, respectively.

## Related work

Considering the rapidly improving restaurant market, a customer’s intention to revisit particular restaurants have been consistently addressed. Because the intention to revisit is one of the most effective predictors of service success, marketing and management primarily focus on intention. For instance, Kumar et al. [14] explored customers’ intention to revisit online food delivery applications through two theoretical frameworks: the stimulus-organism-response and pleasure arousal dominance theories. Based on 446 responses, the mediating roles of pleasure and arousal as well as the indirect determinant of aesthetic formality on revisit intention were confirmed with good fit indices [RMSEA (root mean square error of approximation) = 0.06, CFI (comparative fit index) = 0.92].

Rajput and Gahfoor [15] studied customers’ intention to revisit fast food restaurants through the responses of 433 customers. Considering the conceptual research framework including food/service/environment quality and customer satisfaction, the structural results confirmed the direct motivations of customer satisfaction (0.528) and word of mouth (0.312), indirect factors, and the three quality dimensions.

Han et al. [16] investigated the relationship between revisit intention, perceived satisfaction, consumption emotions, and switching barriers through structural path analysis and qualitative approaches. With 406 validated samples in the United States, the moderating effect of customer satisfaction ($\beta$ = 0.71) and the significant roles of comfort and annoyance on revisit intention were examined.

Meng and Choi [17] introduced a research model that analyzes customer’ behavioral intention to revisit theme restaurants, based on the theory of planned behavior. Based on the results of an on-site survey with 357 customers, it was concluded their attitude and involvement play a mediating role in determining their intention to revisit.

Although several scholars have focused on addressing customer revisit behavior [18], there are several limitations. One such limitation is that there are a huge number of factors, which can have notable impacts on the academic generalization of customer revisits. In addition, it is too difficult to address customer ‘actual revisits’ with traditional data-driven approaches.

Due to the rapidly improved data-driven approaches and technologies for analyzing customer behavior, several recent scholars have addressed the “exact” and ”direct” meanings of customer revisit. Furthermore, recent data analytics, as well as machine and deep learning approaches have allowed researchers to analyze customer revisits. For example, Hwang et al. [11] collected the responses of 133,872 airline service customers, extracted user experience features from customers’ responses, and investigated whether each customer revisits the same airline. User experience dimensions are based on machine learning approaches. They presented that an 83.42% accuracy is achieved in predicting customer revisits in terms of airline services.

Kim and Lee [19] also proposed a systematic framework for predicting customer revisit intention for seven flagship stores. Based on three feature groups—upcoming events, group movements, and store accessibility with approximately 3.75 unique customers in the stores—67–80% accuracy was achieved in predicting customer revisits using the XGBoost classifier.

Kim et al. [20] also proposed a deep neural network framework to address customer repurchase behavior. Considering a 2-year survey including 119,923 (the first round) and 74,088 (the second round) respondents, and the framework integrated by the three sub-modules of long short-term memory (LSTM) (customer comments), convolutional neural network (CNN) (model images), and deep convolutional neural network layers (evaluation rating with brands), 95.13% recall, 94.18% F1-score, 93.25% precision (same brand), and 90.71% accuracy were achieved in predicting whether each customer purchases a smartphone from the same brand.

## Our approach

**Fig. 1:**
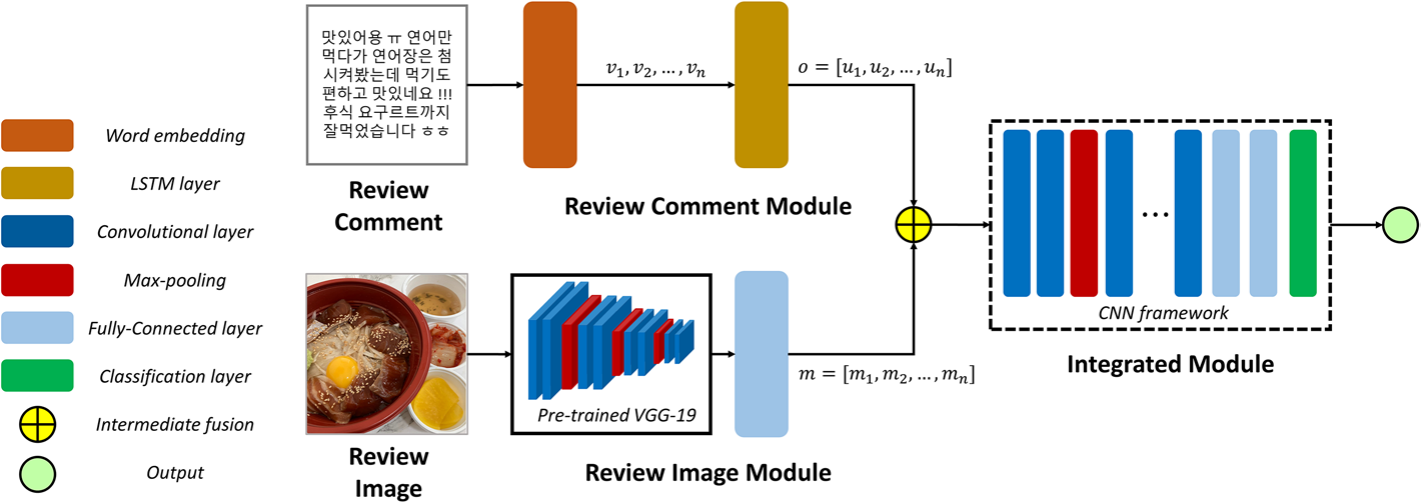
Overview of CRNet

We provide a brief explanation of CRNet, a multimodal deep CNN for customer revisit prediction in MFD services. An overview of CRNet is presented in Fig. 1. CRNet is organized into three modules: (1) a review comment module, (2) a review image module, and (3) an integrated module.

### Review comment module

Each review comment was sequentially embedded with a size of 128. This means that each word in the comment is presented as a single vector, $v_i$. Thus, each comment is presented as $[v_1$, $v_2$, ... $v_n]$, where the length of the comment is denoted by *n*. Then, a LSTM network is employed to present the sequential relationships of each comment with 300 units. The word embedding sequence, $v_i$, and the outcome of the previous unit $u_{i-1}$ from the LSTM units are integrated to present the outcome of word representation, $u_i$. With this procedure, the output of the LSTM network as the text feature, *o*, is presented and denoted as $o = [u_1, u_2, \ldots , u_n]$. This means that unique text feature presentations are used to show the inputs for the proposed model [21].

### Review image module

Each input image is adjusted to $224 \times 224$ for the VGG-19 network, which is pretrained using ImageNet. The output of the VGG network is used as the image feature. Moreover, both $6 \times 6$ regions with 256 vector dimensions are employed for each image. Thus, $m^*$ is presented and organized by $m^*=[m^*_1, m^*_2, \ldots , m^*_n]$. Then, the fully connected layer is added to convert each feature vector into a new vector with the same dimensions as the text feature presentations. Thus, $m=[m_1, m_2, \ldots ,m_n]$ is presented as the image feature matrix [22].

### Integrated module

We used intermediate fusion, which refers to “*the procedures of integrating learned feature representations from intermediate layers of neural networks with features from other modalities as input to a final model*” [23], because this fusion approach shows better performance than other fusion approaches, such as early and late (aggregation) fusions, in this task.

The feature representations extracted from review comments and images are employed as the input to the integrated module. The integrated module is organized into two CNN frameworks. We employed rectified linear units (ReLUs) for all layers without the classification layer. Softmax activation functions are used for the classification layer.

The first CNN framework is composed of 12 convolutional layers ($3\times 3$) with 16 filters and three fully connected layers. It has 128 neurons, and a 0.2 dropout rate. The max-pooling layer ($4\times 4$) is inserted in every two convolutional layers. The second CNN framework includes nine layers: six convolutional layers, two fully connected layers, and a softmax classification layer. The first and last three convolutional layers have 32 and 64 filters, respectively. We employed GridsearchCV to determine the most optimal parameters in 75 epochs, and the Adam optimizer was employed with a learning rate of 0.001.

## Experimental setup

### Datasets

Because only a limited number of scholars have provided datasets for customer revisits, we employed two approaches. First, well-known datasets—which not only address the concept of customer revisits but also consider both images and text features. Thus, the customer repurchase dataset, CRD [20], which includes 74,088 samples (58,175 for repurchases and 15,913 for non-repurchases), was considered. This dataset includes customer review comments and model images, which we employed as review comments and images, respectively.

**Fig. 2:**
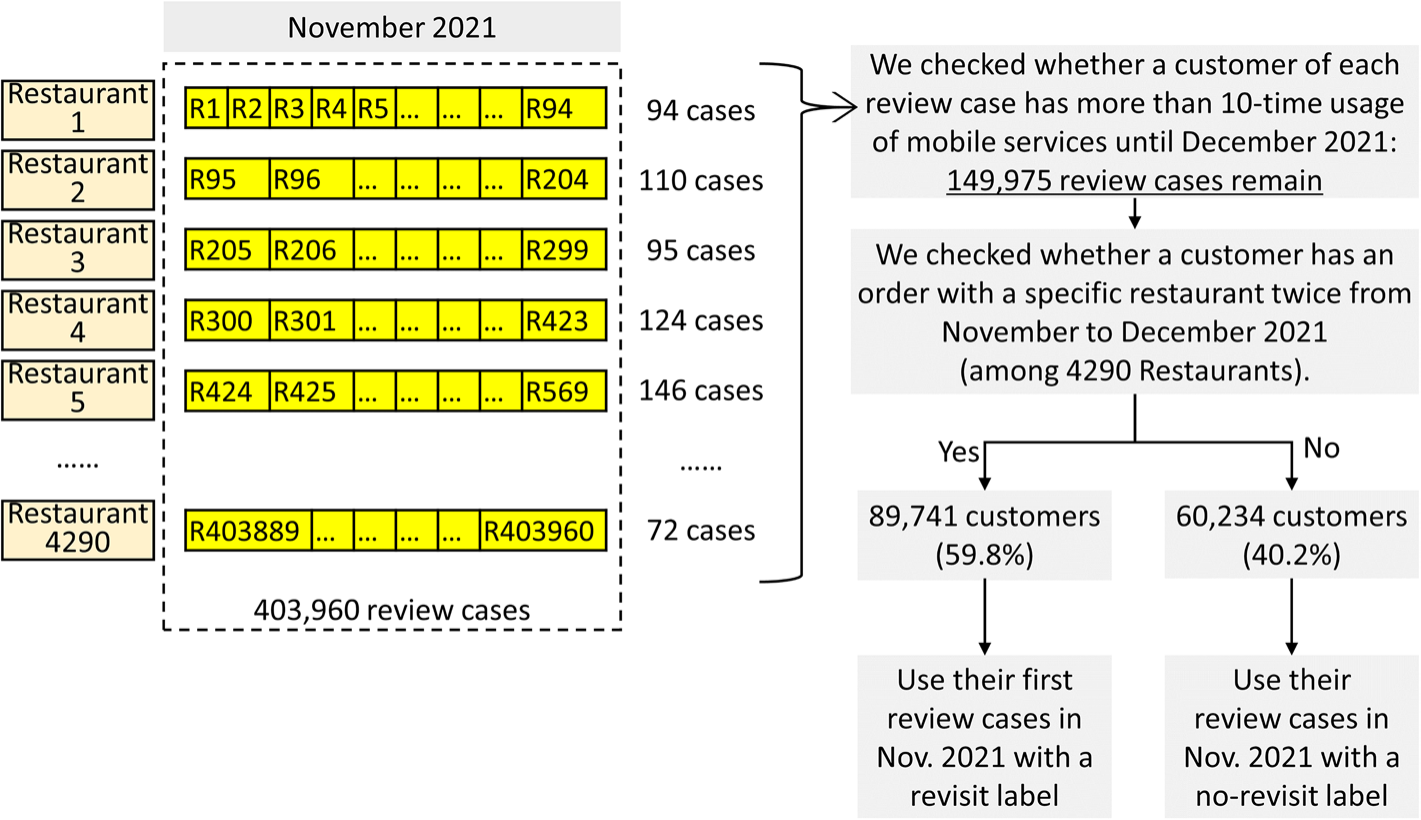
Summary of the data collection and labelling procedures

Second, we aimed to collect a dataset from a popular MFD service in Korea, namely the MFD revisit dataset (MFDRD). Figure 2 shows the summary of the data collection and labelling procedures. We collected 403,960 review cases, which included both review comments with more than 10 words with an image regarding 4290 restaurants in November 2021. Then, in December 31, 2021, we checked each customer who wrote a review, to investigate whether the customer ordered from the same restaurant for more than 10 times.

**Fig. 3:**
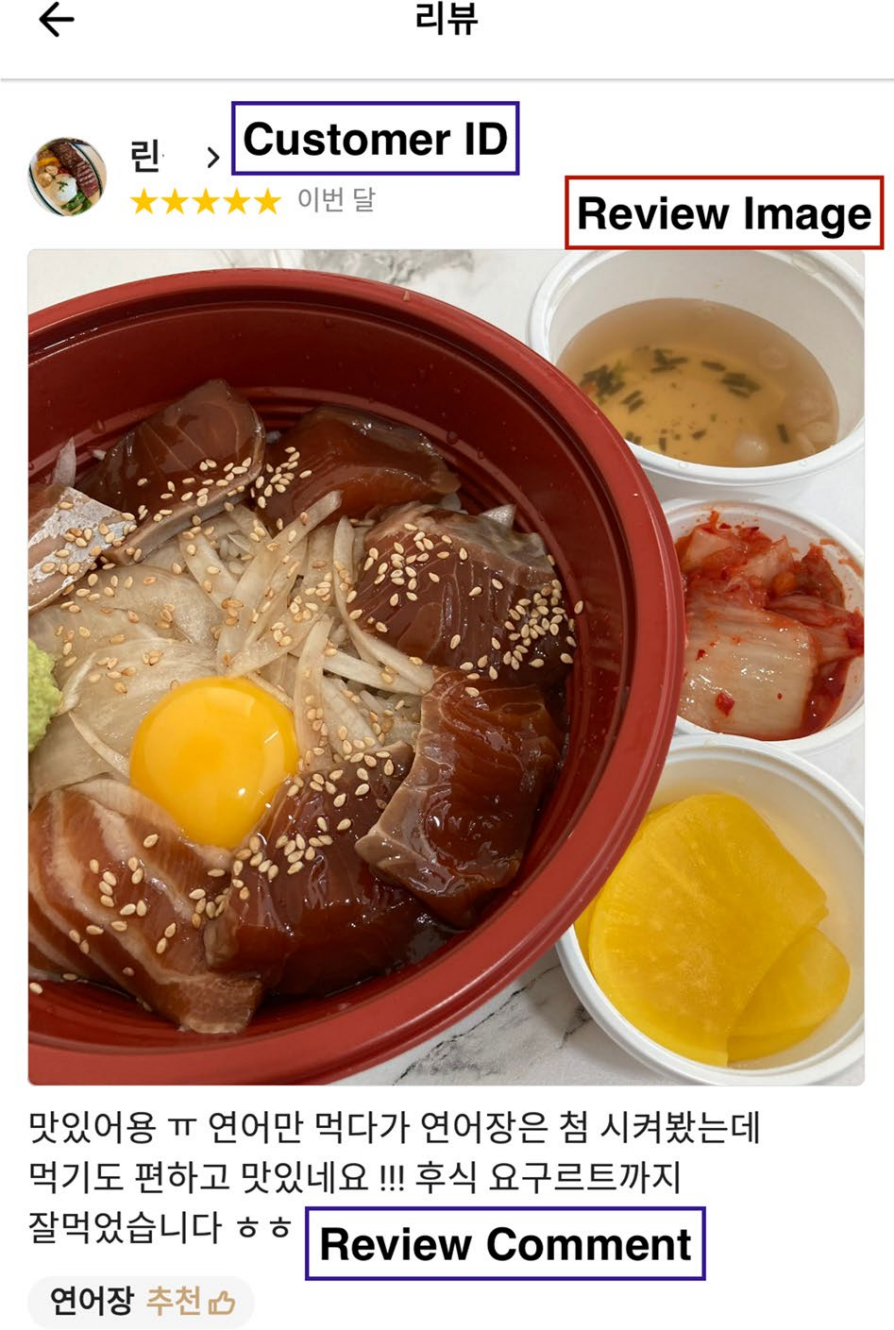
Sample case from the mobile food delivery revisit dataset

Among 403,960 review cases, 149,975 review cases—customers who used a service more than 10 times—were employed. Among them, 89,741 (59.8%) customers revisited the same restaurants; while 60,234 customers (40.2%) did not have a previous order with the restaurant in November 2021. Figure 3 shows an example of the customer review comments and images of MFDs. In the experiment, 60%, 20%, and 20% of the collected cases were employed for training, validation, and testing, respectively. The sample cases are presented in Fig. 3 and in Additional file 1.

### Baseline models

Because our baseline models, as well as CRNet, should address both images and review comments at the same time, both the text-image fusion model [24] and multi-view attentional network model (MVAN) were employed as our baseline models [25].

- Text-image fusion model: an architecture identical to that of a CNN-based text and image fusion model proposed by Gallo et al. [24] was employed. To classify whether each sample is a revisit case, a softmax output layer was attached after the last fully connected layer. Moreover, GridsearchCV was used to find the most optimal parameters (learning rate: 0.01, epoch: 100, ADAM optimizer).
- MVAN: an architecture identical to the MVAN model proposed by Yang et al. [25] was employed, which is organized in the order of feature mapping, interactive learning, and feature fusing modules. To examine a binary revisit classification, the softmax output layer was revised. GridsearchCV was also employed to find the most effective parameters (learning rate: 0.005, epoch: 70, ADAM optimizer).

### Evaluation metrics

We employed four evaluation metrics to test both baseline models and CRNet: *accuracy*, *precision*, *recall*, and *F1-Score*. The following equations were employed to compute *precision*, *recall*, *F1-Score*, and *accuracy*:

$$\text {Precision} = \frac{\text {True Positive (TP)}}{\text {True Positive (TP)} + \text {False Positive (FP)}}$$

$$\text {Recall} = \frac{\text {TP}}{\text {TP}+\text {False Negative (FN)}}$$

$$\text {F1-score} = 2\times {\frac{{\text {Precision}}\times {\text {Recall}}}{\text {Precision}+\text {Recall}}}$$

$$\text {Accuracy} = \frac{\text {TP}+\text {True Negative (TN)}}{\text {TP}+\text {FP}+\text {TN}+\text {FN}}$$

## Experimental results

Table 1 summarizes the experimental results. On the first dataset, CRD, the proposed model, CRNet achieved an accuracy of 95.75%, which is significantly higher than those of the text-image fusion model (74.17%) and MVAN (90.12%). The F1-score showed a similar trend considering the customer revisit class. Considering the characteristics of the proposed research question, the F1-score of CRNet in the revisit class (0.9730) was higher than those of MVAN (0.9378) and the text-image fusion model (0.7825).

**Table 1:** Summary of the results with two datasets

Models	Class	Precision	Recall	F1-Score	Accuracy
Dataset: CRD					
Text-image fusion model	Revisit	0.8753	0.7825	0.8263	0.7417
	Not	0.4266	0.5921	0.4959	
MVAN	Revisit	0.9280	0.9478	0.9378	0.9012
	Not	0.7926	0.7308	0.7605	
CRNet	Revisit	**0.9700**	**0.9760**	**0.9730**	**0.9575**
	Not	0.9102	0.8896	0.8998	
Dataset: MFDRD					
Text-image fusion model	Revisit	0.7725	0.7215	0.7461	0.7065
	Not	0.6231	0.6843	0.6522	
MVAN	Revisit	0.9364	0.9106	0.9098	0.8951
	Not	0.8415	0.8846	0.8747	
CRNet	Revisit	**0.9915**	0.9136	**0.9509**	**0.9436**
	Not	0.8850	**0.9883**	0.9338	

The bold values mean the greatest performance levels

Similar results were also obtained with MFDRD. CRNet (94.36%) achieved higher accuracy than MVAN (89.51%) and the text-image fusion model (70.65%). Moreover, all other metrics of CRNet were higher than those of the other models.

## Discussion and conclusion

In this study, we examined CRNet, a multimodal deep learning framework for predicting customer revisits. Two datasets that address customer revisits were employed in our experiment. In addition, two state-of-the-art multimodal deep learning models for binary classification tasks were employed to evaluate the proposed framework. After developing the text and image modules, the integrated module was attached to determine whether there was a customer revisit.

The experimental results on two datasets, CRD and MFDRD, indicate that the proposed model, CRNet, can effectively examine customer revisit behavior using synchronized multimodal cases—including textual and image contexts. Given three modules and two datasets, we achieved better accuracy and higher scores compared with other baseline models.

From a managerial perspective, considering that the implementation of CRNet is effective in the experiment, service providers, who have multimodal datasets, can easily employ CRNet to explore their customers’ revisit behavior. In addition, consistent with the findings of prior research [21, 26], we confirmed that employing multimodal datasets can present the greater performance than using single-modality datasets in specific tasks.

Although several implications have been provided, notable concerns remain. First, the suggested framework, CRNet, is only effective with synchronized datasets—which are organized by both image and text information. When a partial dataset is provided, the suggested framework cannot be effectively applied. Second, although the employed services in this study do not allow multiple accounts in Korea, there may be different accounts, which can use the actual delivery services. Third, the proposed framework was examined using two datasets. This means that comparable results cannot be obtained on datasets that address customer revisits for other services. Moreover, future research should aim to address other resources for customer revisit information, which can enhance the proposed framework (e.g., metadata information).

## Supplementary Information

**Additional file 1.** Samples of the collected responses.

## Data Availability

The datasets used and/or analysed during the current study are available from the corresponding author on reasonable request.

## Footnotes

1 https://www.prnewswire.com/news-releases/global-online-food-delivery-services-market-report-2021-market-is-expected-to-reach-192-16-billion-in-2025--from-126-91-billion-in-2021---long-term-forecast-to-2030--301285677.html.
